# Acute and Protracted Prenatal Stress Produce Mood Disorder-Like and Ethanol Drinking Behaviors in Male and Female Adult Offspring

**DOI:** 10.3389/fnbeh.2022.862390

**Published:** 2022-05-26

**Authors:** Erbo Dong, Huaibo Zhang, Alison Chu, Subhash C. Pandey

**Affiliations:** ^1^Department of Psychiatry, Center for Alcohol Research in Epigenetics, College of Medicine, Psychiatric Institute, University of Illinois, Chicago, IL, United States; ^2^Jesse Brown VA Medical Center, Chicago, IL, United States

**Keywords:** prenatal maternal stress, SD rats, anxiety, depression, alcohol drinking

## Abstract

**Background:**

Alcohol use disorder (AUD) is a complex and chronic relapsing brain disease, which is often co-morbid with psychiatric disorders such as anxiety and depression. AUD phenotypes differ in men and women. Although genetic factors play an important role in its pathophysiology, epidemiologic evidence suggests that during prenatal development, individuals are more vulnerable to the negative effects of environmental factors that may predispose them to AUD later in life. We explored the effects of prenatal stress on the development of AUD phenotypes as well as anxiety- and depression-like behaviors using rat model.

**Methods:**

In this study, timed-pregnant Sprague Dawley dams were used. Dams in the control group were left undisturbed throughout gestation, whereas dams in stress groups were either subjected to protracted or acute restraint stress under bright light. At adulthood, the anxiety-like, ethanol drinking, and sucrose drinking behaviors were measured using the Light/Dark Box test and two-bottle free-choice procedure.

**Results:**

Compared to the control group, both the male and female offspring in the stress groups exhibited anxiety-like behavior and consumed significantly higher amounts of ethanol in which the acute stress group demonstrated the higher ethanol preference. Moreover, male but not female offspring from the stress groups had decreased sucrose preferences.

**Conclusion:**

These findings suggest that protracted and acute prenatal stress in late pregnancy can induce in anxiety-, depressive-like behaviors, and excessive ethanol intake in adult offspring.

## Introduction

Alcohol use disorder (AUD) is a major public health concern and the third leading preventable disease in the United States (Bouchery et al., 2011; GBD 2016 Alcohol Collaborators, 2018). AUD is a complex and chronic disease that is often accompanied by affective disorders such as anxiety and depression (Koob and Volkow, 2010; Fink et al., 2016), and studies show that patients with depression and anxiety have increased alcohol use compared to their counterparts (Schneier et al., 2010; Wiener et al., 2018). Because of the complex pathogenesis, treatments for the comorbidity of AUD with mood disorders have not been very successful and improved pharmacotherapy is needed in this direction. While AUDs occur in all genders, epidemiological data in the past indicated that the rates of AUD were greater in men than women (Koob and Le Moal, 2001; Baker et al., 2004; Schulte et al., 2009; Yang et al., 2018; Peltier et al., 2019). However, in the past decade, the rates of AUD in women have increased by 84% (Grant et al., 2017; Peltier et al., 2019). There are many factors involved in the increased rates of AUD in women (Guinle and Sinha, 2020; Verplaetse et al., 2021).

Although genetic factors play an important role in AUD pathophysiology, epidemiologic evidence accumulated over decades suggests that individuals during prenatal development may be especially vulnerable to the negative effects of environmental factors that could predispose them to psychiatric disorders, including AUD, later in life (Gordon, 2002; Mulder et al., 2002; Sinha, 2008; Becker et al., 2011; Enoch, 2011; Fine et al., 2014). Previously, in a mouse model using a chronic prenatal restraint stress (PRS) paradigm, we have shown that the adult offspring born from stressed dams developed higher ethanol drinking and anxiety-like behaviors as compared with their counterpart controls (Dong et al., 2018). Studies demonstrate that the impact of prenatal stress on psychological outcomes not only depends on the type and intensity of the stress, but also on the duration of the stress in different gestation periods (Rakers et al., 2013, 2020). Findings from human and animal studies show that the chronic or acute stress during critical phases of gestation activates the maternal hypothalamic–pituitary–adrenal (HPA) axis, inducing impairment of placental catecholamine and maternal infections and thus leading to different phenotypes in the offspring (Rakers et al., 2013, 2020). However, the mechanisms of the maternal transfer of stress to the fetus and the time windows of vulnerability to stress for the fetus remain to be elucidated.

To investigate the relationship between prenatal stress and adult psychopathology, we developed a preclinical model of mild prenatal stress by shortening the duration of stress exposure, focusing on the second, and beginning of the third trimester. Our hypothesis was that prenatal stress at late gestation periods would produce similar behavioral deficits, such as anxiety and excessive ethanol intake, observed in our previously established chronic prenatal stress mouse model (Dong et al., 2018).

## Methods

### Animal and Prenatal Stress

**Figure 1:**
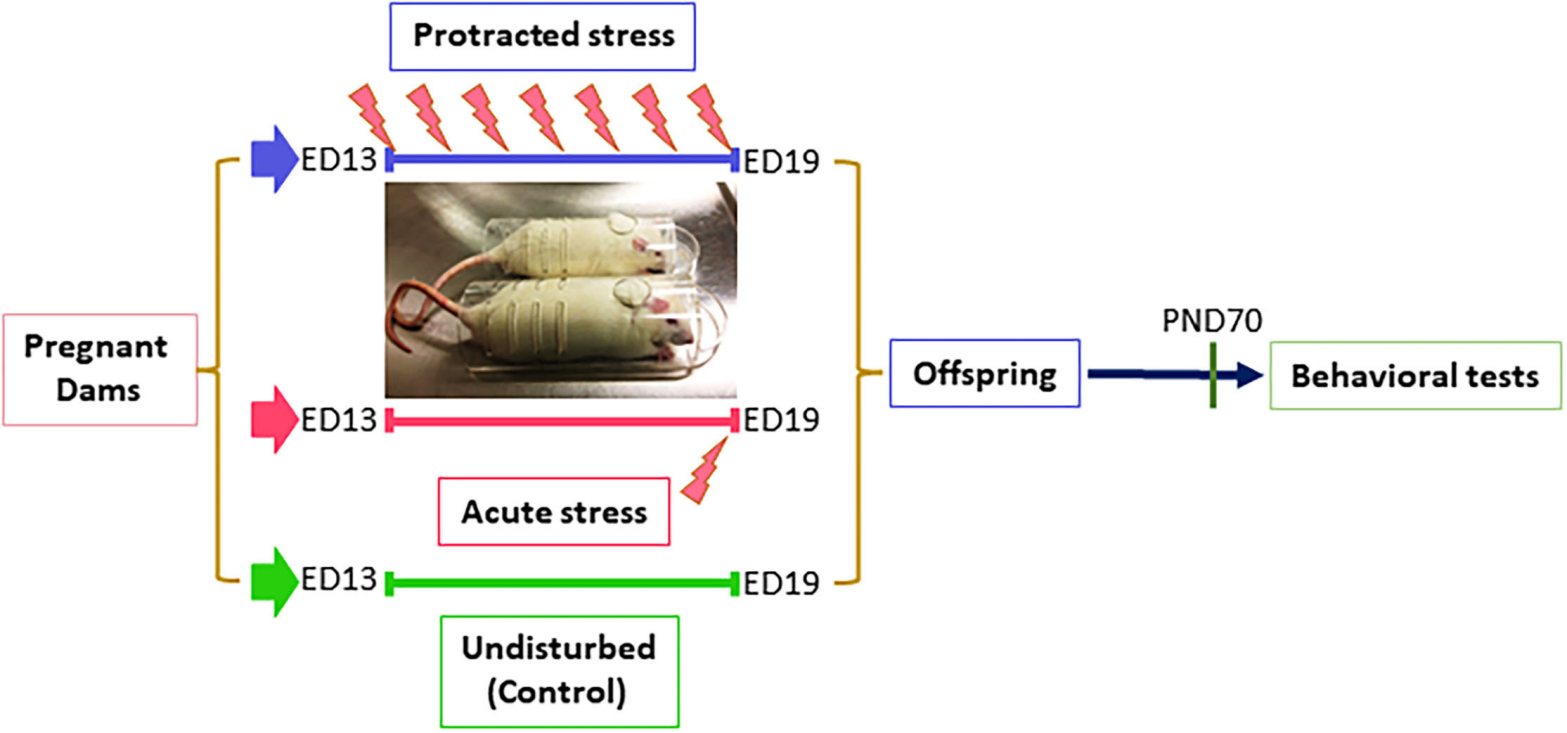
Diagram of stress procedure in this study. Pregnant dams were divided into three groups: control, protracted, and acute stress, in which dams in control group left undisturbed while dams in protracted group were subjected to 7 days repeated restraint stress (twice daily for 30 min) from ED13–19, dams in acute group received a single stress on gestation day 19.

All procedures were performed according to NIH guidelines for animal research (National Research Council (US) Institute for Laboratory Animal Research, 1996) and were approved by the Animal Care Committee of the University of Illinois at Chicago. Timed pregnant (embryonic day 5, ED5) rats (Sprague Dawley) were purchased from the Harlan (Indianapolis, IN), were individually housed with a 12-h light–dark cycle with access to food and water, *ad libitum*. Dams were randomly divided into three groups with two dams in each group. Dams in the control group (non-stressed) were left undisturbed throughout gestation. Dams for prenatal stress were divided into two groups: Group I, subjected to a protracted stress at ED13 and group II, subjected acute stress at ED19. The stress procedure consisted of repeated episodes of restraint in a transparent flat bottom restrainer under a bright light (75 W soft white bulb, about 10 inches above restrainer) for 30 min/twice a day. In this study, the dams in Group I underwent the stress procedure for 7 days total and Group II were stressed for 1 day. After weaning (postnatal day 21, PND21), offspring rats from different dams were mixed and housed separately based on sex. At PND70, the offspring were run through behavioral tests, as described below (Figure 1).

### Behavioral Tests

At PND70, a battery of behavioral tests was performed in the following order: Light/Dark Box (LDB) exploration test, ethanol drinking test, and then sucrose drinking test as described below.

### Light/Dark Box Exploration Test

The procedure was the same as previously described by us (Sakharkar et al., 2014). The computer-controlled LDB apparatus (San Diego Instrument, San Diego, CA) consists of a dark compartment without illumination and a light compartment with illumination (0.25 Amp; light-emitting diode light), and both compartments are connected through an opening. On the day of testing, each rat was allowed a 30 min pretest habituation period in the room before testing. Then, the rat was gently placed in the dark compartment with its head facing away from the opening. The rat was observed for a 5 min test period, and the time spent in each compartment was automatically monitored, recorded, and analyzed by software build. The percentage of time spent in either the dark compartment or light compartment was calculated for each animal. Total ambulation in the light and dark compartments was represented as the general activity of the rat.

### Ethanol Preference Test

Ethanol preference was measured by the two-bottle free-choice paradigm (Pandey et al., 2004). Following a 48-h rest after the LDB test, adult male and female offspring were placed in individual cages with *ad libitum* access to food and water in two bottles and were habituated to drink water from either bottle. Bottle positions were changed daily. Once they were drinking an equal amount of water from either bottle, they were provided with 3% ethanol solution in one bottle and water in the other bottle daily for 3 days, and then concentrations of ethanol were gradually increased to 7% for 3 days, 9% for 3 days, and 12% for 3 days. The consumption of ethanol and water was measured by weighing bottles daily at 6:00 PM, and fresh water and ethanol solution were provided each day at the start of the dark cycle. To prevent liquid spillage, the leak-free sipper tube was used. The mean percentage of ethanol intake and the percentage of water intake were calculated from the daily total fluid intake, and the body weights of rats were measured before and after each dose of ethanol. The ethanol intake was presented as g/kg/day. The ethanol preference was the ratio of EtOH (ethanol) solution intake vs. total fluid intake.

### Sucrose Preference Test

Sucrose preference was measured by the two-bottle free-choice paradigm as described by Wong et al. (1996) and Couceyro et al. (2005). Approximately 7 days after the conclusion of the EtOH drinking test, rats were placed in individual cages with *ad libitum* access to food and water in two bottles, and bottle positions were changed daily. Once the rats started drinking water equally from either bottle, they were provided with 0.2% sucrose solution in one bottle and water in the other bottle daily for 2 days, and then concentrations of sucrose were increased to 2% for 2 days and then 5% for 2 days. The consumption of sucrose and water was measured by weighing bottles daily at 6:00 PM, and fresh water and sucrose solution were provided every day at the start of dark cycle. The sucrose consumption (g/kg/day) was presented as g/kg/day. The sucrose preference was calculated as ratio of sucrose solution intake vs. total fluid intake.

### Statistical Analysis

Significant differences among three groups (control, protracted, and acute) were assessed by 1-way ANOVA for LDB, or 2-way repeated measures ANOVA followed by Bonferroni *post hoc* comparisons for ethanol and sucrose drinking behavior tests using IBM SPSS Statistic 26 (SPSS, Chicago, IL, USA). The values are represented as mean ± S.E.M. The criterion for statistical significance was *p* < 0.05.

## Results

### Anxiety-Like Behaviors in Male and Female Adult Offspring From Prenatally Stressed Dams

**Figure 2:**
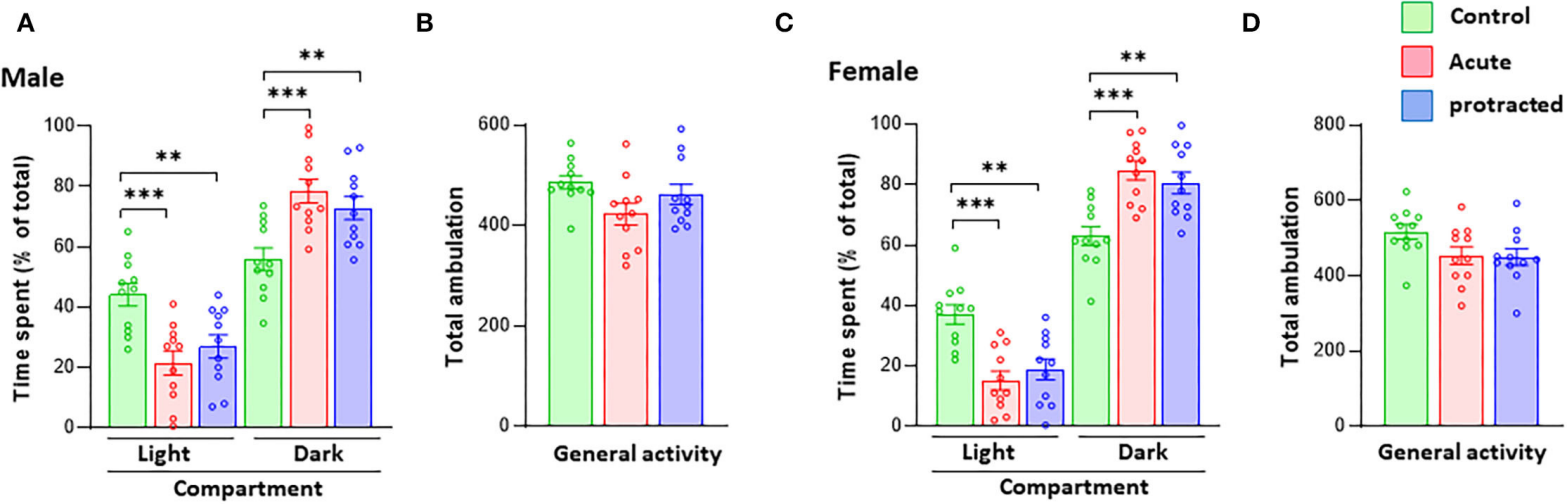
Protracted and acute prenatal stress induces anxiety-like behaviors in young adult offspring at age of postnatal day 70 (PND70). Anxiety-like behaviors were measured using the LDB exploration test. In LDB test, compared with male and female progenies born form non-stressed dams, ones born from dam subjected to protracted or acute stress exhibited high preference to dark compartment in LDB test. **(A,C)** ***p < 0.001 Control vs. Acute, **p < 0.01. There are no statistical differences between protracted and acute groups. All groups showed similar ambulation of LDB exploration **(B,D)** 1-way repeated ANOVA, *n* = 11 for each group). Values are the mean ± Standard Error of the Mean (SEM). Individual value is shown with circle dot on bar diagram.

As reported in previous studies, stress during pregnancy can lead to anxiety-like behaviors in adult offspring mice (Weinstock, 2008, 2016). In this study, using the same stress method but with shorter exposure in pregnant rats, we measured anxiety-like behaviors in adult offspring born from mothers subjected to protracted and acute stress during pregnancy. In the LDB test, as shown in Figure 2A, male offspring rats from both protracted and acute groups spent significantly more time in the dark than the light compartment as compared to their counterpart non-stressed controls [time in dark compartment: *F*_(2, 30)_ = 9.403, *p* = 0.0007; 1-way ANOVA, *n* = 11]; [time in light compartment: *F*_(2, 30)_ = 9.476, *p* = 0.0006; 1-way ANOVA, *n* = 11]; there were no statistical differences between protracted and acute groups. The total ambulation in LDB of either Group I or Group II rats did not significantly differ from control rats (Figure 2B) [*F*_(2, 30)_ = 2.848, *p* = 0.0737, 1-way ANOVA, *n* = 11], showing no change in the general activity of the offspring rats. Similar results were found in the female rats (Figures 2C,D) [time in dark compartment: *F*_(2, 30)_ = 12.32, *p* = 0.0001; time in light compartment: *F*_(2, 30)_ = 12.86, *p* = 0.0001; total ambulation: *F*_(2, 30)_ = 3.015, *p* = 0.064, 1-way ANOVA, *n* = 11]. This suggests that both protracted and acute prenatal stress can lead to the development of anxiety-like behaviors in both male and female adult offspring.

### Ethanol Drinking Behaviors in Male and Female Adulthood Offspring From Prenatally Stressed Dams

**Figure 3:**
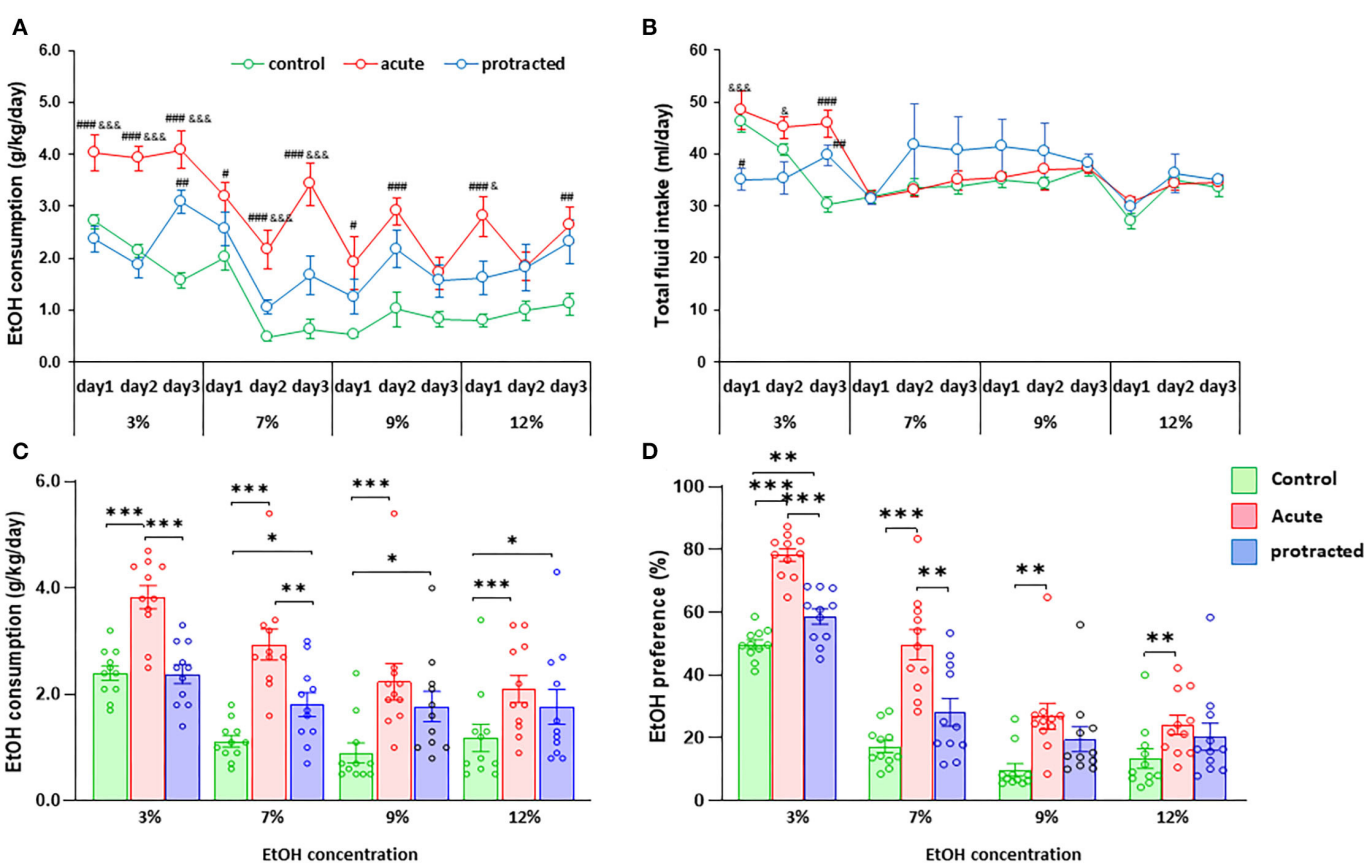
Protracted and acute prenatal stress-induced excessive ethanol intake in young adult male offspring at age of PND70. Ethanol (EtOH) drinking behavior was measured using two-bottle free-choice test. In **(A,C)**, a daily and means of 3-day ethanol consumption at given concentrations with corresponding control groups shows that male rats born from dams subjected to either protracted or acute stress developed significantly high ethanol consumption and preference **(D)** characterized by excessive ethanol intake (g/kg/day) to all four concentrations of ethanol provided and less water intake. *p < 0.05, **p < 0.01, ***p < 0.001: Protracted, Acute vs. Control; ^#^*p* < 0.05, ^##^*p* < 0.01, ^###^*p* < 0.001: Protracted, Acute vs. Control; ^&^*p* < 0.05, ^&&^*p* < 0.01, ^&&&^*p* < 0.001: Acute vs. Protracted. **(B)** Shows total fluid intake in three groups. 2-way repeated ANOVA, *n* = 11 for each group. Values are the mean ± SEM. Individual value is shown with circle dot on bar diagram.

Our previous report demonstrated that the chronic prenatal stress could cause more ethanol consumption in young adult offspring in mice (Dong et al., 2018). To explore whether protracted or acute prenatal stress leads to behavioral alterations in ethanol drinking, we next assessed the drinking behaviors in these rats using a two-bottle free-choice paradigm. Strikingly, when pharmacologically relevant concentrations of 3–12% ethanol were offered, male offspring from stressed groups voluntarily consumed higher amounts of ethanol compared to their control counterparts [*F*_(2, 30)_ = 25.303; *p* = 0.00004, 2-way repeated ANOVA, *n* = 11 per group]. Figures 3A,C show the daily and mean 3-day ethanol intake at each concentration per group. Interestingly, there was a much more robust ethanol intake in adult male offspring in the acute stress group than the protracted group. Although fluid intake differed between groups at the beginning of drinking paradigm (3% ethanol), there were no further significant differences in total fluid intake among the three groups [*F*_(2, 30)_ = 0.834; *p* = 0.444, 2-way repeated ANOVA, *n* = 11] (Figure 3B). The drinking behavior of offspring rats born from protracted and acute stressed dams is characterized by increased intake of ethanol solution and decreased water intake [*F*_(2, 30)_ = 24.388, *p* = 5.1389E-7, 2-way repeated ANOVA, *n* = 11] (Figure 3D).

**Figure 4:**
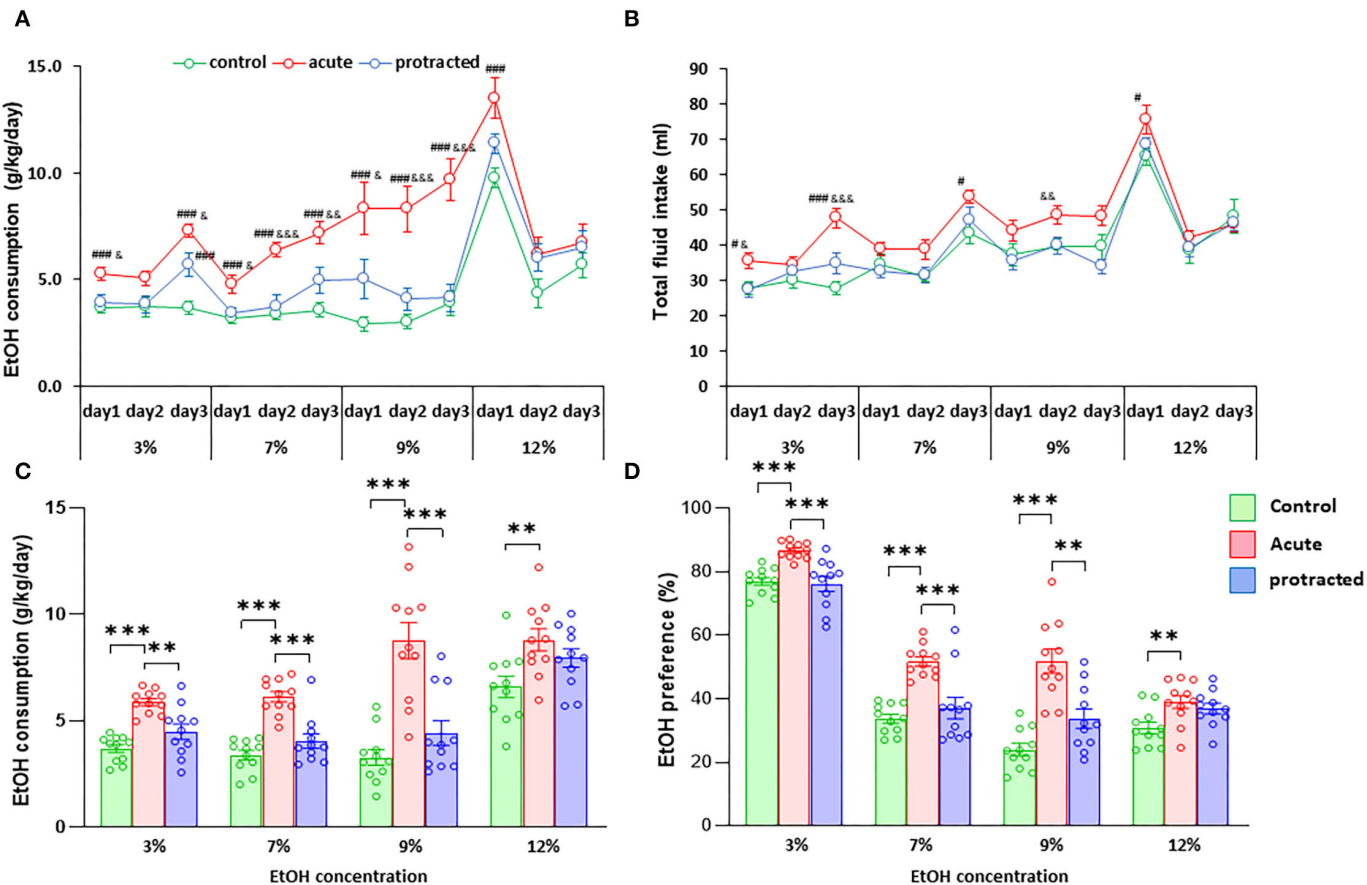
Protracted and acute prenatal stress-evoked excessive ethanol intake in young adult female offspring at age of PND70. Ethanol drinking behavior was measured using two-bottle free-choice test. In **(A,C)**, a daily and means of 3-day ethanol consumption at given concentrations with corresponding control groups shows that male rats born from dams subjected to either protracted or acute stress developed significantly high ethanol consumption and preference **(D)** characterized by excessive ethanol intake (g/kg/day) to all four concentrations of ethanol provided and less water intake. *p < 0.05, **p < 0.01, ***p < 0.001: Protracted, Acute vs. Control; ^#^*p* < 0.05, ^##^*p* < 0.01, ^###^*p* < 0.001: Protracted, Acute vs. Control; ^&^*p* < 0.05, ^&&^*p* < 0.01, ^&&&^*p* < 0.001: Acute vs. Protracted. **(B)** Shows total fluid intake in three groups. 2-way repeated ANOVA, *n* = 11 for each group. Values are the mean ± SEM. Individual value is shown with circle dot on bar diagram.

**Figure 5:**
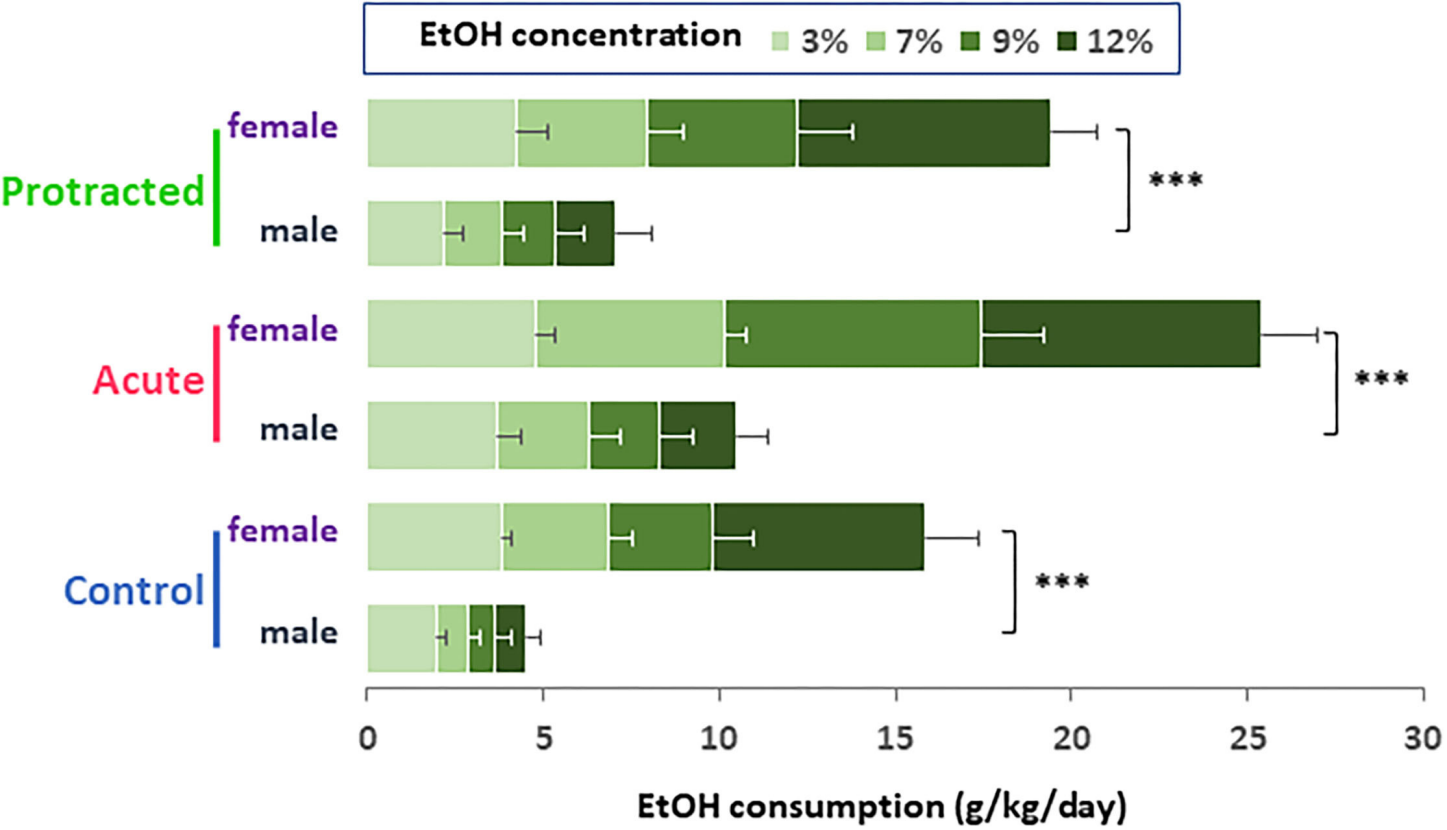
Bar diagram showing differences in ethanol intake between male and female rats. Female offspring rats in control group (non-stressed) consumed much higher ethanol than their male counterparts. Either protracted or acute prenatal stress enhanced the sex differences in ethanol drinking (male vs. female rats, ***p < 0.001, 2-way ANOVA, *n* = 11 for each group). Values are the mean ± SEM.

Similar findings were observed in the female offspring. As shown in Figures 4A,C, the stressed groups demonstrated significantly higher ethanol consumption as compared with the control group [*F*_(2, 30)_ = 37.28; *p* = 0.000001, 2-way repeated ANOVA, *n* = 11 per group]. Among the three groups, the offspring from the acute stress group exhibited the most robust ethanol intake, which was associated with higher total fluid intake as compared with the protracted and control groups [*F*_(2, 30)_ = 14.307; *p* = 0.00004, 2-way repeated ANOVA, *n* = 11 per group] (Figure 4B). As shown in Figure 4D, female offspring rats from stressed groups demonstrated a significant ethanol preference as compared with the control group [*F*_(2, 30)_ = 44.668, *p* = 1.0121E-9, 2-way repeated ANOVA, *n* = 11]. In addition, we found that female rats consumed much more ethanol overall than their male counterparts. Figure 5 shows the sex differences in ethanol intake between male and female rats in which female offspring rats demonstrated a much higher preference to ethanol than male rats at all concentrations of ethanol offered (sex x animal: *F*_(2, 60)_ = 3.378; *p* = 0.041, 2-way repeated ANOVA, *n* = 11 per group). It was also realized that the female rats in the control group (non-stressed) consumed more ethanol than their male counterparts and prenatal stress synergized their preference to ethanol. These findings provide the evidence that mild prenatal stress, especially the acute one, can significantly lead to excessive ethanol consumption in adult male and female offspring.

### Physical Anhedonia in Male and Female Adulthood Offspring From Prenatally Stressed Dams

**Figure 6:**
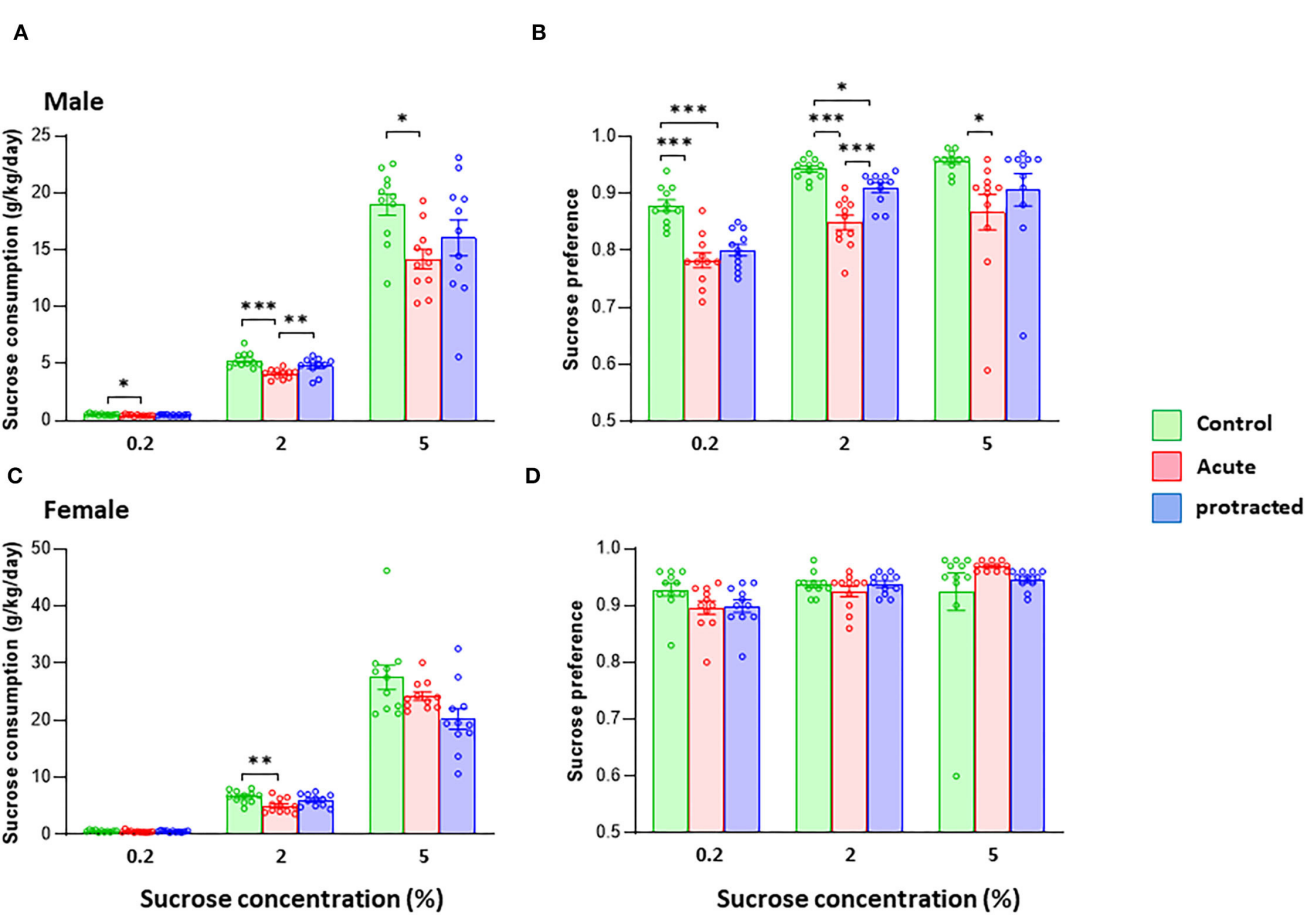
Protracted and acute prenatal stress induces anhedonia-like behaviors in young male and female adult offspring at age of postnatal day 70. The sucrose consumption was measured using two-bottle free-choice paradigm. **(A,B)** Present that the sucrose intake (g/kg/day) of the tests for both male and female rats. The consumption **(A)** and preference **(B)** to the 3 given concentrations of sucrose were significantly decreased in male rats from protracted and acute groups, in which the rats in acute group exhibited the most significant reduction as compared with control group, *p < 0.05, **p < 0.01, ***p < 0.001: Protracted, Acute vs. Control; ^#^*p* < 0.05, ^##^*p* < 0.01: Acute vs. Control. In female rats, acute stress group showed significant less in sucrose consumption **(C)** only at 2% sucrose. There was no difference in sucrose preference among three groups **(D)**, 2-way repeated ANOVA followed by Bonferroni test, *n* = 11 for each group. Values are the mean ± SEM. Individual value is shown with circle dot on bar diagram.

It has been shown that the exposure to stress during pregnancy can change the trajectory of neuronal system development, leading to psychiatric disorders such as depression later in life (Markham and Koenig, 2011). One key symptom of major depression is reduction or loss of hedonic motivation. Here, we examined whether protracted and acute prenatal stress evoked physical anhedonia in offspring. In animal studies, a sucrose preference test is often used to mimic anhedonia behaviors (Liu et al., 2018). We measured sweet preference with a two-bottle free-choice paradigm using three concentrations of sucrose. As shown in Figures 6A,B, male rats born from dams subjected to both protracted and acute stress exhibited remarkable reduction in sucrose intake and reduced sucrose preference, with the rats in the acute group exhibiting the most significantly reduced sucrose consumption at all concentrations offered when compared with protracted and control groups [male, *F*_(2, 30)_ = 6.735; *p* = 0.004, 2-way repeated ANOVA, *n* = 11 per group]. Figure 6B shows decreased preference found in the offspring of stressed groups [*F*_(2, 30)_ = 19.9982, *p* = 0.000003, 2-way ANOVA, *n* = 11].

In female offspring, although protracted and acute groups demonstrated less sucrose consumption, the differences among groups were not large enough to reach statistical significance [*F*_(2, 30)_ = 2.475; *p* = 0.101, 2-way repeated ANOVA, *n* = 11 per group] (Figures 6C,D). There was no statistical significance in sucrose preference among the three groups [*F*_(2, 30)_ = 411, *p* = 0.667, 2-way repeated ANOVA, *n* = 11]. These results demonstrate that the mild prenatal stress can reduce the sucrose preference in adult offspring, especially the males.

## Discussion

Offspring from prenatal stress dams exhibit several behavioral phenotypes (Weinstock, 2008, 2016). To explore the underlying molecular mechanisms for these phenotypes, we established a preclinical mouse model using chronic stress paradigm in which dams were subjected to restraint stress three times a day combined with bright light at ED7 until delivery. We observed that the young adult offspring of stressed pregnant dams exhibited anxiety-like behaviors and higher ethanol intake as compared to non-stressed controls, reflecting some symptoms seen in AUD subjects. These findings are similar to data showing that stress induces anxiety like behavior (Fride and Weinstock, 1988) which leads to increase alcohol seeking and consumption behavioral responses (Becker et al., 2011; Noori et al., 2014).

Because the impact of prenatal stress on psychological outcomes not only depends on the type and intensity of the stress but also depends on the duration of the exposure (Rakers et al., 2013, 2020), it is necessary to explore whether mild stressor and a shorter duration of exposure leads to similar behavioral changes observed in the chronic mouse model. In the present study, we modeled prenatal stress in two ways: protracted stress, starting at ED13 for 7 days, and acute stress, with a single stress exposure at ED19. These gestation periods are equivalent to the second trimester and the beginning of the third trimester of human fetal development (Charil et al., 2010; Patten et al., 2014). These developmental periods are critically important for brain growth, and stress during these periods may exert a long-term impact on the brain development in progeny, leading to significant behavioral deficits. Instead of mice, we selected rats for evaluating these stress conditions because the rat is the most often used species in neuroscience (Ellenbroek and Youn, 2016) and found that the prenatal stress produces ethanol drinking and anxiety-like behaviors in male and female adult offspring in both rats and mice.

Like our previous observations in chronic prenatal stress mouse model (Dong et al., 2018), both protracted and acute prenatal stress during the second trimester and the beginning of the third trimester led to anxiety-like behaviors and significant ethanol intake in both adult male and female offspring at all concentrations of ethanol provided. Strikingly, both male and female rats in the acute stress group showed the strongest voluntarily ethanol consumption compared with both protracted stress and control groups. These findings provide evidence that even a single event of acute stress during pregnancy can induce remarkably excessive ethanol drinking and heightened anxiety in both male and female adult progeny. Since the ethanol intake was measured in 24 h access paradigm, we were not certain if animals drank enough to reach intoxication levels. Since alcohol intoxication is one of symptoms of AUD, more experiments such as limited access (2 or 4 h) of ethanol intake and measurement of blood ethanol concentration need to be performed to characterize further this model.

Interestingly, we observed a sex difference in ethanol intake characterized by higher baseline drinking in female rats than in male rats, and prenatal stress led to higher ethanol preference in female offspring. From other animal studies, it was found that female rats acquired the self-administration of ethanol more rapidly (Koob, 2003). According to recent reports, the rates of AUD onset for women compared to men have increased by 84% (Grant et al., 2017; Peltier et al., 2019). Increasing evidence suggests that the changes in the levels of sex hormones and neurosteroids in the certain brain structures may be associated with sex difference in alcohol consumption (Muti et al., 1998; Peltier et al., 2019; Verplaetse et al., 2021). Our behavioral data suggest that this rodent model might be a useful preclinical model to explore the sex difference in AUDs. Therefore, our future studies will explore the roles of sex hormones (such as estradiol and testosterone), neurosteroids (such as progesterone), their metabolites, and receptor expression in ethanol consumption in male and female rodents.

There is a high level of concurrent and sequential comorbidity between anxiety and depression in young adults (Garber and Weersing, 2010; Van Lieshout and Boylan, 2010). Anhedonia, a deficit of hedonic activity defined by a reduction of motivation and anticipatory pleasure, is a major component of depressive disorders. To investigate whether mild prenatal stress induces depression-like behaviors, we measured sucrose preference as a proxy for reduced hedonic activity. In a two-bottle free-choice test with sucrose, we observed that mild prenatal stress, especially within the acute stress group, significantly decreased sucrose consumption in male rats. This provides evidence that, along with anxiety-like behaviors, both protracted and acute prenatal stress can cause depression-like behavior. The differences in sucrose intake between female treatment groups were not large enough to reach statistical significance, suggesting that males may be more vulnerable to mild prenatal stressors. It is interesting to point out that the anhedonia-like behavior observed may not be due to prenatal stress alone. Although the sucrose preference test was performed 7 days after the ethanol drinking test, it is possible that ethanol deprivation effects (Heyser et al., 1997, 1998; Funk et al., 2004; Vengeliene et al., 2014) may have affected sucrose intake. Therefore, the results may be related to both effect of ethanol exposure and prenatal stress and further experiments are needed to examine prenatal stress effects directly on sucrose intake.

The mechanism by which prenatal stress produces adult psychopathology is less clear but may be related to epigenetic reprogramming, as we reported earlier in mouse model (Dong et al., 2018). Furthermore, it has been shown that the stress during gestation can affect mothers in several ways: (i) increase in cortisol (corticosterone) level, which activates the maternal HPA axis, (ii) increase in catecholamine and cytokine concentrations. These maternal changes can be transferred through impaired placental to the fetus leading to aberrant development of central nerve system (Rakers et al., 2013, 2020). We have reported that the behavioral deficits in offspring caused by prenatal stress were associated with remarkable reduction of dendritic spine density in the frontal cortex accompanied by the decreased expression of key synaptic molecules (Dong et al., 2018). In the present study, the possible mechanisms underlying acute prenatal stress that produces more robust behavioral outputs in offspring is not clear and required further study. Here, it is possible that dams may develop resilience to repeat the patterns of chronic stress, which may counteract the impacts of the stressors.

In conclusion, findings from this study indicate that gestation during a late period is vulnerable to mild physical stressors such as restraint stress and bright light. Stress during these periods can lead to anxiety-, depression-like phenotypes and excessive ethanol intake in adult male and female offspring. However, several important studies are necessary to validate this model, such as the time-window of vulnerability to stressors, AUD-related behaviors such as stress-coping, cognition, ethanol seeking, and withdrawal symptoms as well as underlying neural and molecular mechanisms. Furthermore, it is essential to study the effects of prenatal stress on dams and mechanisms by which maternal behavioral changes affect the fetus biology and adult psychopathology. Nonetheless, the present study provides a useful preclinical model for investigating the pathogenesis of alcohol use disorders caused by prenatal stress.

## Data Availability Statement

The original contributions presented in the study are included in the article/supplementary material, further inquiries can be directed to the corresponding author.

## Ethics Statement

The animal study was reviewed and approved by Office of Animal Care and Institutional Biosafety Committee, University of Illinois at Chicago.

## Author Contributions

ED designed and discussed experimental plan with SCP. ED ran the experiments with HZ and AC. ED analyzed data and prepared all figures. ED and SCP interpreted findings and wrote the manuscript. All authors contributed to the article and approved the submitted version.

## Funding

This study was supported by the NIH-NIAAA grant R21AA027848 to ED and P50AA022538, UO1AA019971 and by the VA Senior Research Career Scientist award to SCP.

## Conflict of Interest

The authors declare that the research was conducted in the absence of any commercial or financial relationships that could be construed as a potential conflict of interest.

## Publisher's Note

All claims expressed in this article are solely those of the authors and do not necessarily represent those of their affiliated organizations, or those of the publisher, the editors and the reviewers. Any product that may be evaluated in this article, or claim that may be made by its manufacturer, is not guaranteed or endorsed by the publisher.
